# Durable response to venetoclax, azacytidine, and ruxolitinib in chronic-phase myelofibrosis resistant to ruxolitinib: a case report and literature review

**DOI:** 10.3389/fonc.2025.1644661

**Published:** 2025-08-08

**Authors:** Xiaohong Liu, Xiujuan Zhang, Hongxia Shi

**Affiliations:** ^1^ Department of Hematology, Yangquan First People’s Hospital, Yangquan, Shanxi, China; ^2^ Peking University People’s Hospital, Peking University Institute of Hematology, National Clinical Research Center for Hematologic Disease, Beijing, China

**Keywords:** myelofibrosis, ruxolitinib, drug-resistant, venetoclax, azacytidine, treatment

## Abstract

Myelofibrosis (MF) is a philadelphia chromosome-negative chronic myeloprolifera- tive neoplasm.It has a worse prognosis than polycythemia vera and essential thrombocy- themia.At present,both Chinese and foreign guidelines recommend ruxolitinib as first-line treatment for IPSS/DIPSS/DIPSS-Plus patients with splenomegaly in intermediate-risk-2 and high-risk MF-CP.However,long-term follow-up revealed possible increase in the discontinuation rate of ruxolitinib due to drug resistance and other reasons with the prolongation of the treatment duration. At this study,it imposes a great challenge for clinical management owing to the lack of standard treatment for patients who have lost efficacy due to drug resistance,and the absence of subsequent treatment strategies. Significantly, venetoclax combined with azacytidine, with or without the use of ruxolitinib, was reported to be effective and safe for patients in MF-accelerated/acute phase.Here, we report a case of MF-CP patient who was unresponsive to ruxolitinib, with therapeutic response after applying venetoclax and azacytidine in combination with ruxolitinib. After treatment, the patient showed improved condition. After two courses of treatment with venetoclax and azacytidine combined with ruxolitinib, the patient has been continuously treated with oral ruxolitinib and has achieved a therapeutic effect for more than 1 year. The treatment response of this patient we reported provides a new safe and effective treatment method for MF-CP patients who are no longer responsive to ruxolitinib.

## Introduction

Myelofibrosis(MF) is a philadelphia chromosome-negative chronic myeloproliferative neoplasm characterized by anemia, leucocytosis or hypoplasia, thrombocytosis or hypo- plasia, hepatosplenomegaly, constitutional symptoms, and fibrous tissue proliferation in the bone marrow. As a heterogeneous myeloproliferative phenotype, MF has varied survival time, ranging from several months to several years. MF has a worse prognosis than polycythemia vera and essential thrombocythemia. At present, the only solution for this condition is allogeneic hematopoietic stem cell transplantation, yet accompanied by high treatment-related mortality and transplant- related complications (1).

According to COMFORT-1 and COMFORT-2 studies, Ruxolitinib was effective in reducing spleen size, alleviating MF, and improving the quality of life in MF-chronic phase (CP) patients from moderate-risk-2 and high-risk groups; and it can significantly prolong overall survival (OS), with a median OS of 5.3 years (2–4). Both Chinese and foreign guidelines recommend ruxolitinib as first-line treatment for IPSS/DIPSS/DIPSS-Plus patients with splenomegaly in intermediate-risk-2 and high-risk MF-CP (1, 5). However, long-term follow-up revealed possible increase in the discontinuation rate of ruxolitinib due to drug resistance and other reasons with the prolongation of the treatment duration (6, 7). For these patients the NCCN guideline recommend treatment with Fedratinib, Pacritinib or Momelotinib (5). However, in China, none of these drugs are available.

Significantly, v enetoclax combined with azacytidine, with or without the use of ruxolitinib, was reported to be effective and safe for patients in MF-accelerated/acute phase (8–10). To our knowledge, for MF-CP patients who are no longer responsive to ruxolitinib, there is no report on the effectiveness of combined therapy using venetoclax (VEN) and azacytidine (AZA) with ruxolitinib (RUX). Here is our report of one MF-CP patient who was unresponsive to ruxolitinib, with therapeutic response after applying venetoclax and azacytidine in combination with ruxolitinib. This is an exploratory study. This case report may provide a new safe and effective strategy for clinical management.

## Case presentation

In April 2018, a 55-year-old female patient experienced discomfort in the left abdomen. A complete blood count (CBC) showed anaemia, with a white blood cell count of 3.8 × 10^9^ cells/L, hemoglobin of 97 g/L, and platelet count of 160 × 10^9^ cells/L, with no visible circulating blasts in the peripheral blood. Abdominal ultrasound: splenomegaly (intercostal thickness of about 7.1cm, length and diameter of about 23cm), and hepatomegaly. The bone marrow cytology and flow cytometry showed normal proliferation, without abnormal phenotypes or blast cells. Chromosomal analysis showed a normal karyotype. Gene detection in bone marrow: BCR-ABL fusion gene negative, and JAK2V617F positive. Bone marrow biopsy indicated reticular fibers (+), significant fibrous tissue proliferation in the medullary cavity(MF grade 2), visible myeloid and erythroid cells, increased number of megakaryocytes, and strange nuclei with excessive lobulation of megakaryocytes. Based on these examinations, the patient was diagnosed with myeloproliferative neoplasm, MF-CP(IPSSintermediate-risk-2,DIPSS intermediate- risk-2, and DIPSS Plus intermediate-risk-2), with a MPN10 score of 22 points. The patient had medical history of coronary atherosclerotic heart disease. On 26 April 2018, the patient was treated with ruxolitinib 15mg twice per day orally. Subsequently, the patient’s symptoms have improved and the spleen shrinks. However, the patient presented with aggravated anemia, with hemoglobin at 76g/L. Later, thalidomide, danazole, prednisone and erythropoietin were added for treatment anemia, and aspirin to prevent thrombosis. Meanwhile, corresponding scheme of medications was adjusted according to regular review of the CBC, liver and kidney function, as well as liver and spleen color Doppler ultrasound. After treatment, the patient showed stable disease, with gradual shrinkage of the spleen and stabilized blood cell count. The color Doppler ultrasound in January 2022 showed abnormality of splenomegaly (intercostal thickness and long diameter of about 5.5cm and 19cm, respectively), with no enlargement of the liver (portal vein 1.0cm).

**Figure 1:**
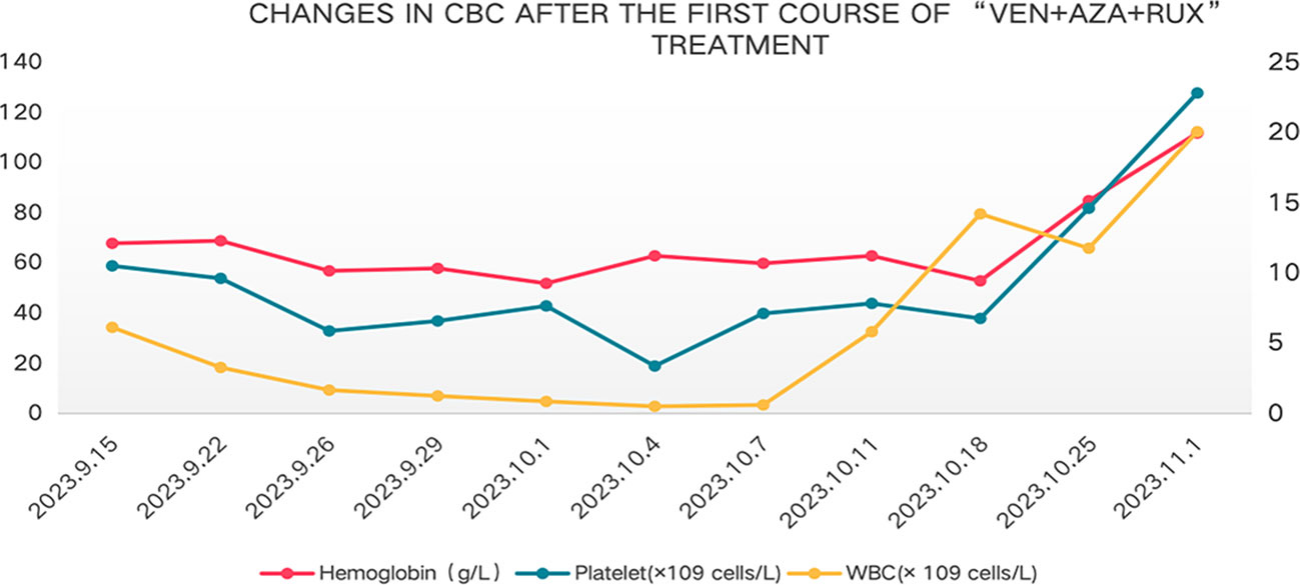
Changes in CBC after the first course of “VEN+AZA+RUX” treatment.

In August 2023, the patient showed poor appetite, distension in the left abdomen, and discomfort. CBC showed white blood cell count of 2.4×10^9^ cells/L, hemoglobin of 82g/L, and platelet count of 40 × 10^9^ cells/L.MPN10 score was 33 points. Abdominal ultrasound revealed large liver, with a maximum oblique diameter of about 16.0cm in the right lobe; with splenomegaly, intercostal thickness of about 7.6cm, length diameter of about 23cm. It is considered that ruxolitinib treatment has lost its efficacy. Literature reports that ruxolitinib discontinuation syndrome (RDS) occurred in 13.5% patients after a median time of 7 days from ruxolitinib stop. And in multivariable Cox regression analysis, platelet count <100 × 10^9^ cells/L and spleen≥10 cm below costal margin at ruxolitinib stop were significantly associated with higher probability of RDS (11). On this basis, the patient was treated with ruxolitinib gradually adjusted to 5mg/d. On 15 September 2023, the patient was tested with white blood cell count of 6.15 × 10^9^ cells/L, hemoglobin of 68 g/L, and platelet count of 59 × 10^9^ cells/L, with the presence of 2% circulating blasts in the peripheral blood. Blood erythropoietin was 96.7mIU/ml (reference range of 4.30-29.00). Abdominal ultrasound revealed large liver, with an anterior posterior diameter of the left lobe of about 10.7cm and a maximum oblique diameter of about 16.8cm in the right lobe; with splenomegaly, intercostal thickness of about 7.9cm, length diameter of about 25cm, splenic portal vein dilation, widest inner diameter of about 2.3cm, and splenic infarction. Cytology of bone marrow indicated reduced proliferation of nucleated cells, and no primitive immature cells. Flow cytometry showed no abnormal phenotypes or blast cells. Chromosomal analysis showed a complex karyotype: 46,XX,der(1;6)(q10;q10)[10]/46, idem, add(3) (p25) [9]/46,XX,del(20) (q11.2)[1]. MDS FISH of bone marrow showed 20q positive in 8.5% and negative in the remaining part. Bone marrow biopsy suggested extremely reduced proliferation, no increase in immature cells, no megakaryocytes, and visible focal fibrosis (MF focal grade 2). Next-generation sequencing showed mutation of Tier I:JAK2 V617F(VAF85%);mutation of Tier II: ARID2 R1677*(VAF45%),BCORL1 G777* (VAF2.7%).The diagnosis of MF-CP(IPSS high-risk,DIPSS intermediate-risk-2, DIPSS Plus high-risk,MIPSS70 high-risk and MIPSS70-plus very-high-risk) remained in this patient. MPN10 score was 48 points. In China, drugs Fedratinib, Pacritinib and Momelotinib are all unavailable. After thorough communication with the patient and her family, exploratory treatment with venetoclax, azacitidine and ruxolitinib was administered. On 15 Septem- ber 2023, the patient was provided with venetoclax (100mg, quaque die (qd),days1–7), azacytidine (100mg,qd,days1–7) and ruxolitinib (5mg,qd,continuously orally). However, after combined treatment, the patient developed grade IV bone marrow suppression (granulocytopenia for 7 days, and platelet count of <20×10^9^ cells/L for 1 day) (**Figure 1**), pulmonary infection, herpes zoster. The patient improved and was discharged after receiving symptomatic supportive treatment such as anti-infection, anti-virus, and component blood transfusion (1 unit of platelet collection via transfusion irradiation machine, and 10 units of leukocyte suspension red blood cell removal). On 1 November 2023, 40 days after combined therapy, the patient’s CBC was white blood cell count of 20.10 × 10^9^ cells/L, neutrophil count of 16.10 × 10^9^ cells/L, hemoglobin of 112 g/L, and platelet count of 128 × 10^9^ cells/L. Abdominal ultrasound showed normal size and shape of the liver, and widened inner diameter of the main portal vein, with a maximum width of about 1.4cm; enlarged spleen, with a thickness of about 7.8cm and a length of about 21.5cm; and widened diameter of the splenic portal vein, with a maximum width of about 1.5cm. MPN10 score of the patient was 16 points. All these results indicated an effective treatment.On 17 November 2023,the patient was given the second course of “venetoclax + azacytidine+ ruxolitinib”. On 4 January 2024, the patient had white blood cell count of 12.30 × 10^9^ cells/L, hemoglobin of 97 g/L, and platelet count of 59 × 10^9^ cells/L. Due to the patient’s personal reasons, the subsequent “venetoclax + azacytidine”treatment was not continued, but ruxolitinib(5-15mg,twice a day,adjusted according to platelet count) was continued oral. On April 16, 2024,143 days after the second therapy, the patient had white blood cell count of 5.60 × 10^9^ cells/L, hemoglobin of 100 g/L, and platelet count of 97 × 10^9^ cells/L. Abdominal ultrasound displayed full shape of the liver, with the inner diameter of the main portal vein widened by 1.6cm, splenomegaly, intercostal thickness of about 7.0cm,length of about 20.2cm,and widened inner diameter of splenic portal vein, with a maximum width of about 1.6cm. On 19 November 2024,12 months after the second chemotherapy, the patient received another reexamination via abdominal ultrasound, showing full shape of the liver, with widened inner diameter of the main portal vein by 1.6cm; as well as splenomegaly, with intercostal thickness of about 7.2cm,and length and diameter of about 22.5cm. In addition, CBC suggested white blood cell count of 22.56×10^9^ cells/L, hemoglobin of 109g/L, and platelet count of 118×10^9^ cells/L. The bone marrow cytology showed normal proliferation, 3% primitive immature cells. Flow cytometry showed 1.56% blast cells. Chromosomal analysis showed a abnormal karyotype:46,XX,add(6)(p25)[16]/46,idem,add(3)(p25)[3]/46,XX[1]. Bone marrow biopsy suggested extremely reduced proliferation, no increase in immature cells, and visible focal fibrosis (MF focal grade 3).Next-generation sequencing showed mutation of Tier I:JAK2 V617F(VAF95.4%);muta- tion of TierII: ARID2 R1677* (VAF45.7%), BCORL1 G777* (VAF31.9%).

## Discussion

MF is characterized by splenomegaly, as well as severe systemic symptoms including extreme fatigue, shortness of breath, nighttime sweating, weight loss, fever, and bone pain, usually with JAK2, CALR, or MPL mutations (12). MF includes PMF, post-ET MF, and post-PV MF, eventually progressing to acute leukemia in approximately 10%~20% of MF cases (13). Based on peripheral blood and bone marrow blasts, MF comprises of several sub-phenotype such as MF-CP, -accelerated phase, and -blast phase. Ruxolitinib has been recommended as the first-line option for IPSS/DIPSS/DIPSS -Plus intermediate-2 and high-risk MF-CP patients with splenomegaly. However, the outcome is unsatisfactory owing to the increased discontinuation rate resulted from prolonged treatment time. Francesca Palandri et al. reported that the 1-year, 2-year and 3-year discontinuation rates of ruxolitinib in MF-CP patients were 22.2%, 32.4% and 40.8%, respectively, due to lack of response, loss of efficacy, disease progression and intolerance (6). CN Harrison et al. reported a 5-year discontinuation rate of 73.3% for patients receiving ruxolitinib (7). For these MF patients who are intolerant or no longer unresponsive, there is also limited and challenged subsequent treatment.

As a selective BCL-2 inhibitor, venetoclax can induce apoptosis by selectively inhibiting BCL-2, leading to the release of cytochrome C from mitochondria (14). It has been approved by the U.S. FDA for the treatment of acute myeloid leukemia in 2018. Azacytidine may synergistically inhibit the pro-survival MCL1- and BCL-XL based pathways, thereby increasing the dependence of myeloid malignancies (AML, MDS and MPNs) cells on BCL-2 (15, 16). Venetoclax in combination with hypomethylating agents is effective and safe for both *de novo* and relapsed/refractory acute myeloid leukemia. According to existing clinical studies (17, 18), venetoclax-azacitidine regimen can realize a composite complete remission rate of 55%~75% in the treatment of newly diagnosed elderly AML patients. Meanwhile, for relapsed and refractory AML, venetoclax combined with hypomethylating agents also exhibits satisfactory efficacy, with an objective response rate of 60%-64% and a median duration of leukemia-free status of 8.8-8.9 months (19, 20). This scheme can also be effective in patients with relapsed refractory AML who have previously used hypomethylating agents, with a CR/CRi rate of 43% and a median OS time of 10.8 months for CR/CRi patients (21).

Nowadays, there is no standard treatment for MF-accelerated/acute phase. Hypomethylating agents in combination with JAK inhibitors or with venetoclax are common therapeutic options with confirmed efficacy and safety in clinical work. In a multicenter retrospective study, Naseema Gangat et al. reported the use of venetoclax combined with demethylating drugs (azacitidine/decitabine) for treating 32 patients with MPN-BP, including 9 patients with previous PMF, 12 with post-ET MF, and 11 with post-PV MF, with a median age of 69 years. The CR+CRi rate was 44%, and the median OS was 8 months (8). Another retrospective study on the treatment of MPN-BP with venetoclax combined with demethylating drugs (azacitidine/decitabine) involved 2 cases of previous PMF, 2 cases of post-ET MF, 3 cases of post PV-MF, 3 cases of ET, 1 case of PV, and 1 case of MPN-u. The median age was 71 years (48–81), and the total effective rate (CR+PR) was 42% (9). In another study on five MF-BP patients [median age of 76 years (72-84)] treated with azacytidine, venetoclax and ruxolitinib, Thomas Systchenko et al. reported a total response rate of 80% and a complete response rate of 40%, as well as a median follow-up of 10 months and a median OS of 13.4 months, with post-treatment improvement in patients’ quality of life. The treatment was effective and safe without serious adverse reactions (10).

COMFORT-I study suggested that ruxolitinib was effective and safe for intermediate -2 and high-risk MF through 5-year long-term follow-up. This scheme could rapidly and continuously shrink the spleen, significantly improve survival, and reduce the risk of death (22). Ruxolitinib combined with azacytidine has potential synergy for spleen length reduction and BM fibrosis improvement. In a Phase II clinical trial of the combination treatment of ruxolitinib and azacitidine in 46 patients with MF who hadn’t received prior therapy with RUX or AZA,62% and 71% of these patients had a reduction in subcostal spleen length greater than 50% at 24 weeks and at any time during the study, respectively. At 24 months, 57% of patients experienced improved grade of myeloreticular fibrosis, supporting the efficacy and safety of this combined therapy for MF patients (23). For the MF patients who are intolerant or no longer unresponsive to ruxolitinib, the NCCN guideline recommend treatment with Fedratinib, Pacritinib or Momelotinib (5). Fedratinib is a potent and selective JAK2 and FLT3 inhibitor. The phase II non- randomized JAKARTA-2 trial showed that fedratinib was also effective in reducing splenomegaly and symptom burden in patients with ruxolitinib-resistant intermediate-2- risk/high-risk MF (24). However, in China, none of these drugs are available, no effective strategies for subsequent treatment are available for MF patients who are intolerant to or unresponsive to ruxolitinib. Navitoclax (ABT-263) is an oral BCL-2 inhibitor with a high affinity for BCL-XL, BCL-2, and BCL-W. Claire N. Harrison et al. conducted a Phase II multicenter, open-label clinical trial on 34 patients with intermediate-to-high-risk MF-CP who had failed or progressed after ruxolitinib treatment. With the use of ruxolitinib combined with Navitoclax, 26.5% of patients achieved SVR35 at 24 weeks and 41% at any time during the study, with a median duration of 13.8 months. At 24 weeks, 30% of patients achieved TSS50, 33% of patients had grade 1–2 BMF improvements, and 64% of patients showed improved anemia, yet without the achievement of median OS. The most common adverse event was thrombocytopenia (25).

**Figure 2:**
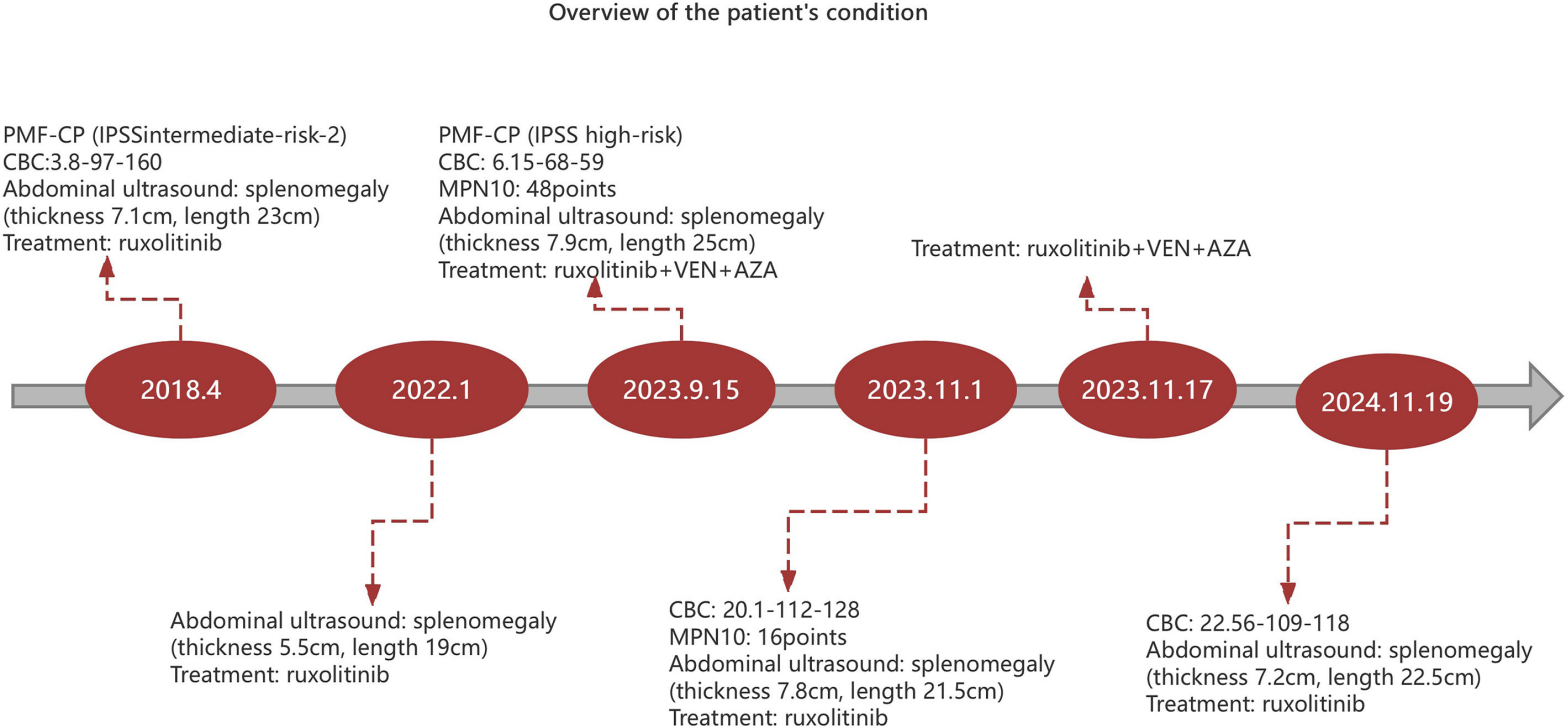
Overview of the patient’s condition. CBC (complete blood count): The following order is white blood cells (×10^9^ cells/L), hemoglobin (g/L) and platelets(×10^9^ cells/L).AZA, azacytidine. VEN, venetoclax.

In our case, the patient was diagnosed with MF-CP (IPSS intermediate-risk-2, DIPSS intermediate-risk-2, and DIPSS Plus intermediate-risk-2) in April 2018. The patient had improved condition after treatment with ruxolitinib, with decreased MPN10 score, and alleviated splenomegaly compared to before. The efficacy lasted for over 5 years. In August 2023, the patient showed changes in the condition, including poor tolerance, discomfort with left abdominal distention, as well as progressive decline of hemoglobin and platelets. With a possibility of disease progression, the patient was discovered to be still in the MF-CP after evaluation of bone marrow. It was speculated that the patient was no longer responsive to ruxolitinib. After one course of treatment with a combined therapy of venetoclax, azacytidine, and ruxolitinib, the patient was observed with improved general condition, hematopoietic recovery, hemoglobin and platelet levels returned to normal, smaller spleen than before,and decreased MPN10 score. The treatment was effective. During the treatment, the patient developed grade IV bone marrow suppression, pulmonary infection, and herpes zoster. After treatment, the patient showed improved condition. After two courses of treatment with venetoclax and azacytidine combined with ruxolitinib, the patient has been continuously treated with oral ruxolitinib and has achieved a therapeutic effect for more than 1 year. The treatment response of this patient we reported provides a new safe and effective treatment method for MF-CP patients who are no longer responsive to ruxolitinib (**Figure 2**). In addition, elderly patients with MF had poor tolerance to chemotherapy and a longer bone marrow suppression time. The treatment was evaluated to be ineffective in hematology and spleen one month after the first chemotherapy. However, by day 40, there was a significant improvement in hematology and a significant reduction in spleen size compared to before.

## Conclusion

This is an exploratory study and the first report of safe and effective treatment of a patient with MF-CP who failed to respond to ruxolitinib by venetoclax and azacitidine combined with ruxolitinib. The usage of venetoclax and azacitidine in patients with MF-CP may be different from that in patients with acute leukemia. The time for response evaluation in patients with MF-CP may be different from that in patients with acute leukemia. In our case, the evaluation is required around 6 weeks after treatment, and consolidation therapy is also beneficial for condition management and CBC. However this finding also has limitations. This is a single case with multiple prior treatments and the duration of response may not be generalizable. In the future, further well-designed clinical trials are needed for confirmation.

## Data Availability

The original contributions presented in the study are included in the article/supplementary material. Further inquiries can be directed to the corresponding author.

## References

[1] Leukemia and Lymphoma Group, Chinese Society of Hematology, Chinese Medical Association. Chinese guideline on the diagnosis and treatment of primary myelofibrosis (2019). Chin J Hematol. (2019) 40:1–7. doi: 10.3760/cma.j.issn.0253-2727.2019.01.001, PMID: 30704220 PMC7351691

[2] Verstovsek S Mesa RA Gotlib J Levy RS Gupta V DiPersio JF. A double-blind, placebo-controlled trial of ruxolitinib for myelofibrosis. N Engl J Med. (2012) 366:799–807. doi: 10.1056/NEJMoa1110557, PMID: 22375971 PMC4822164

[3] Harrison C Kiladjian JJ Al-Ali HK Gisslinger H Waltzman R Stalbovskaya V. JAK inhibition with ruxolitinib versus best available therapy for myelofibrosis. N Engl J Med. (2012) 366:787–98. doi: 10.1056/NEJMoa1110556, PMID: 22375970

[4] Verstovsek S Gotlib J Mesa RA Vannucchi AM Kiladjian JJ Cervantes F. Long-term survival in patients treated with ruxolitinib for myelofibrosis: COMFORT-I and -II pooled analyses. J Hematol Oncol. (2017) 10:156. doi: 10.1186/s13045-017-0527-7, PMID: 28962635 PMC5622445

[5] NCCN clinical practice Guidelines in oncology Myeloproliferative Neoplasms Version 2.2024. Available online at: http://www.nccn.org (Accessed October 2024).

[6] Palandri F Breccia M Bonifacio M Polverelli N Elli EM Benevolo G. Life after ruxolitinib: Reasons for discontinuation, impact of disease phase, and outcomes in 218 patients with myelofibrosis. Cancer. (2020) 126:1243–52. doi: 10.1002/cncr.32664, PMID: 31860137

[7] Harrison CN Vannucchi AM Kiladjian JJ Al-Ali HK Gisslinger H Knoops L. Long-term findings from COMFORT-II, a phase 3 study of ruxolitinib vs best available therapy for myelofibrosis. Leukemia. (2016) 30:1701–7. doi: 10.1038/leu.2016.148, PMID: 27211272 PMC5399157

[8] Gangat N Guglielmelli P Szuber N Begna KH Patnaik MM Litzow MR. Venetoclax with azacitidine or decitabine in blast-phase myeloproliferative neoplasm: A multicenter series of 32 consecutive cases. Am J Hematol. (2021) 96:781–9. doi: 10.1002/ajh.26186, PMID: 33844862 PMC8251544

[9] Gangat N Morsia E Foran JM Palmer JM Elliott MA Tefferi A. Venetoclax plus hypomethylating agent in blast-phase myeloproliferative neoplasm: preliminary experience with 12 patients. Br J Haematol. (2020) 191:e120–4. doi: 10.1111/bjh.17084, PMID: 32945528

[10] Systchenko T Chomel JC Gallego-Hernanz P Moya N Desmier D Maillard N. Combination of azacitidine, venetoclax and ruxolitinib in blast phase myeloproliferative neoplasms. Br J Haematol. (2023) 202:284–8. doi: 10.1111/bjh.18853, PMID: 37183377

[11] Palandri F Palumbo GA Elli EM Polverelli N Benevolo G Martino B. Ruxolitinib discontinuation syndrome: incidence, risk factors, and management in 251 patients with myelofibrosis. Blood Cancer J. (2021) 11:4. doi: 10.1038/s41408-020-00392-1, PMID: 33414394 PMC7791065

[12] Tefferi A. Primary myelofibrosis: 2023 update on diagnosis, risk-stratification, and management. Am J Hematol. (2023) 98:801–21. doi: 10.1002/ajh.26857, PMID: 36680511

[13] Zhang Y Zhou H Duan M Gao S He G Jing H. Safety and efficacy of jaktinib (a novel JAK inhibitor) in patients with myelofibrosis who are intolerant to ruxolitinib: A single-arm, open-label, phase 2, multicenter study. Am J Hematol. (2023) 98:1588–97. doi: 10.1002/ajh.27033, PMID: 37470365

[14] Guerra VA DiNardo C Konopleva M. Venetoclax-based therapies for acute myeloid leukemia. Best Pract Res Clin Haematol. (2019) 32:145–53. doi: 10.1016/j.beha.2019.05.008, PMID: 31203996 PMC6581210

[15] Bogenberger JM Delman D Hansen N Valdez R Fauble V RA M. Ex vivo activity of BCL-2 family inhibitors ABT-199 and ABT-737 combined with 5-azacytidine in myeloid malignancies. Leuk Lymphoma. (2015) 56:226–9. doi: 10.3109/10428194.2014.910657, PMID: 24707940 PMC4331188

[16] Bogenberger JM Kornblau SM Pierceall WE Lena R Chow D Shi CX. BCL-2 family proteins as 5-Azacytidine-sensitizing targets and determinants of response in myeloid malignancies. Leukemia. (2014) 28:1657–65. doi: 10.1038/leu.2014.44, PMID: 24451410 PMC4131248

[17] DiNardo CD Jonas BA Pullarkat V Thirman MJ Garcia JS Wei AH. Azacitidine and Venetoclax in Previously Untreated Acute Myeloid Leukemia. N Engl J Med. (2020) 383:617–29. doi: 10.1056/NEJMoa2012971, PMID: 32786187

[18] DiNardo CD Pratz K Pullarkat V Jonas BA Arellano M Becker PS. Venetoclax combined with decitabine or azacitidine in treatment-naive, elderly patients with acute myeloid leukemia. Blood. (2019) 133:7–17. doi: 10.1182/blood-2018-08-868752, PMID: 30361262 PMC6318429

[19] Aldoss I Yang D Aribi A Ali H Sandhu K Al Malki MM. Efficacy of the combination of venetoclax and hypomethylating agents in relapsed/refractory acute myeloid leukemia. Haematologica. (2018) 103:e404–7. doi: 10.3324/haematol.2018.188094, PMID: 29545346 PMC6119155

[20] Zappasodi P Brociner M Merati G Nizzoli ME Roncoroni E Boveri E. Venetoclax and azacytidine combination is an effective bridge to transplant strategy in relapsed/refractory acute myeloid leukemia patients. Ann Hematol. (2021) 100:1111–3. doi: 10.1007/s00277-020-04333-7, PMID: 33175198

[21] Ram R Amit O Zuckerman T Gurion R Raanani P Bar-On Y. Venetoclax in patients with acute myeloid leukemia refractory to hypomethylating agents-a multicenter historical prospective study. Ann Hematol. (2019) 98:1927–32. doi: 10.1007/s00277-019-03719-6, PMID: 31187237

[22] Verstovsek S Mesa RA Gotlib J Gupta V DiPersio JF Catalano JV. Long-term treatment with ruxolitinib for patients with myelofibrosis: 5-year update from the randomized, double-blind, placebo-controlled, phase 3 COMFORT-I trial. J Hematol Oncol. (2017) 10:55. doi: 10.1186/s13045-017-0417-z, PMID: 28228106 PMC5322633

[23] Masarova L Verstovsek S Hidalgo-Lopez JE Pemmaraju N Bose P Estrov Z. A phase 2 study of ruxolitinib in combination with azacitidine in patients with myelofibrosis. Blood. (2018) 132:1664–74. doi: 10.1182/blood-2018-04-846626, PMID: 30185431 PMC6265645

[24] Harrison CN Schaap N Vannucchi AM Kiladjian JJ Tiu RV Zachee P. Janus kinase-2 inhibitor fedratinib in patients with myelofibrosis previously treated with ruxolitinib (JAKARTA-2): A single-arm, open-label, non-randomised, phase 2, multicentre study. Lancet Haematol. (2017) 4:e317–24. doi: 10.1016/S2352-3026(17)30088-1, PMID: 28602585 PMC8207822

[25] Harrison CN Garcia JS Somervaille TCP Foran JM Verstovsek S Jamieson C. Addition of Navitoclax to Ongoing Ruxolitinib Therapy for Patients With Myelofibrosis With Progression or Suboptimal Response: Phase II Safety and Efficacy. J Clin Oncol. (2022) 40:1671–80. doi: 10.1200/JCO.21.02188, PMID: 35180010 PMC9113204

